# Supplementary material for: Metformin and Niclosamide Synergistically Suppress Wnt and YAP in APC-Mutated Colorectal Cancer
Source: Cancers (Basel). 2021 Jul 9;13(14):3437. doi: 10.3390/cancers13143437 (PMC8308039; doi:10.3390/cancers13143437)
Supplement: Supplementary file 1 [file cancers-13-03437-s001.zip › cancers-1228604-supplementary.pdf]

Supplementary Materials

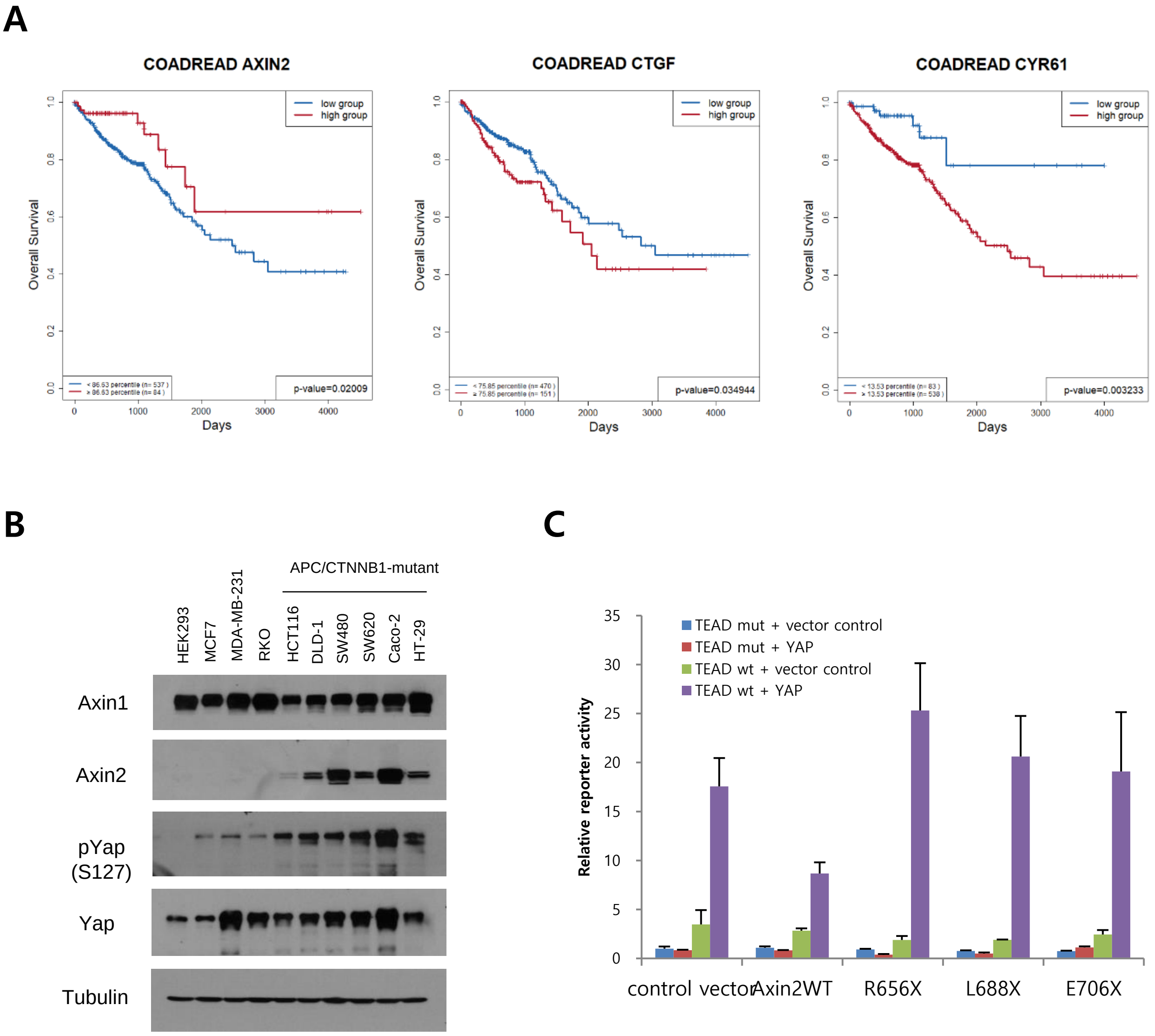

Figure S1. Axin2 activates Hippo pathway in APC-mutated CRC cells.

A Kaplan–Meier survival graphs for colorectal cancer patients on the basis of Axin2, CTGF and CYR61 transcript abundances at an optimal threshold indicated by percentile numbers. Samples with high abundance of Axin2, CTGF and CYR61 are represented with red lines. A log-rank test was used to calculate statistical significances.

B Total Axin1, Axn2, YAP and p127-YAP abundance from various human cancer cell lines according to APC/CTNNB1 mutational status.

C Axin2 overexpression suppressed TEAD transcriptional activity in a Axin2 C-terminus dependent manner. Relative TEAD reporter activity was measured expressing indicated vectors in 293 cells.

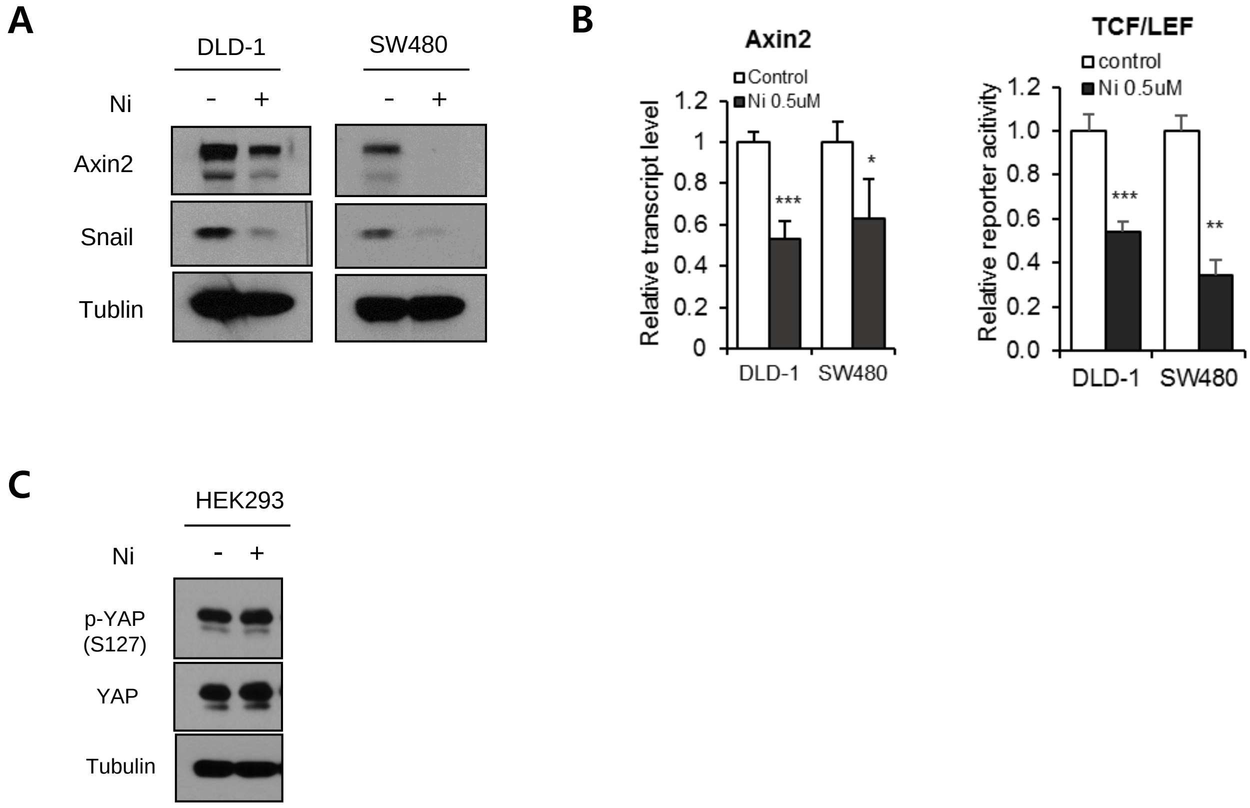

Figure S2. Niclosamide suppresses canonical Wnt activity and Snail abundance in APC-mutated CRC cells.

A The APC-mutant CRC cells were treated with niclosamide (0.5 μM), and immunolot analysis to determined Axin2 and Snail abundance.

B Niclosamide suppresses canonical Wnt transcriptional activity measured by Axin2 transcript abundance (left) and TCF/LEF reporter activity in CRC cells.

C The protein levels of YAP and p-YAP were measured in 293 cells treated with niclosamide.

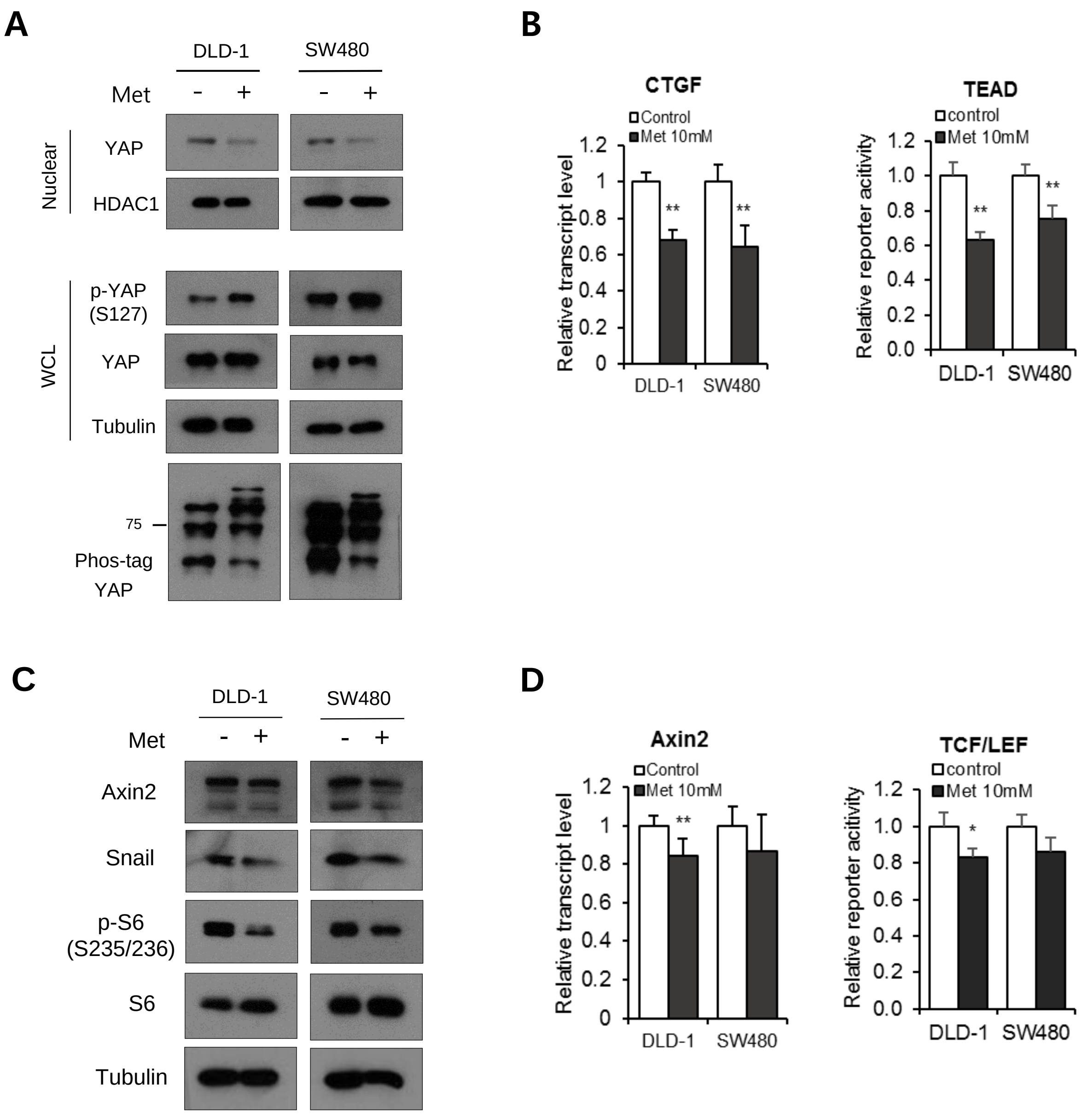

Figure S3. Metformin suppresses nuclear YAP activity.

A,B The CRC cells were treated with metformin (10 mM) and YAP phosphorylation status in nuclear fraction and whole cell lysate (WCL) was determined by p-S127-YAP antibody and mobility shift on a phos-tag gel (a). Relative CTGF transcript level (left) and relative TEAD reporter activity (right) were determined by qRT-PCR for CTGF and TEAD reporter assay, respectively (b). Statistical significances compared to control was denoted as **, P < 0.01 by a two-tailed Student's t-test.

C CRC cells were treated with metformin 10mM for 16h and immunoblot analysis of endogenous Axin2, Snail, p-S235/236-ribosomal protein S6 protein abundance.

D Relative Axin2 transcript abundance (left) and TCF/LEF reporter (Top flash) activity (right) in CRC cells, respectively. Statistical significances compared to control was denoted as *, P < 0.05; **, P < 0.01 by a two-tailed Student's t-test.

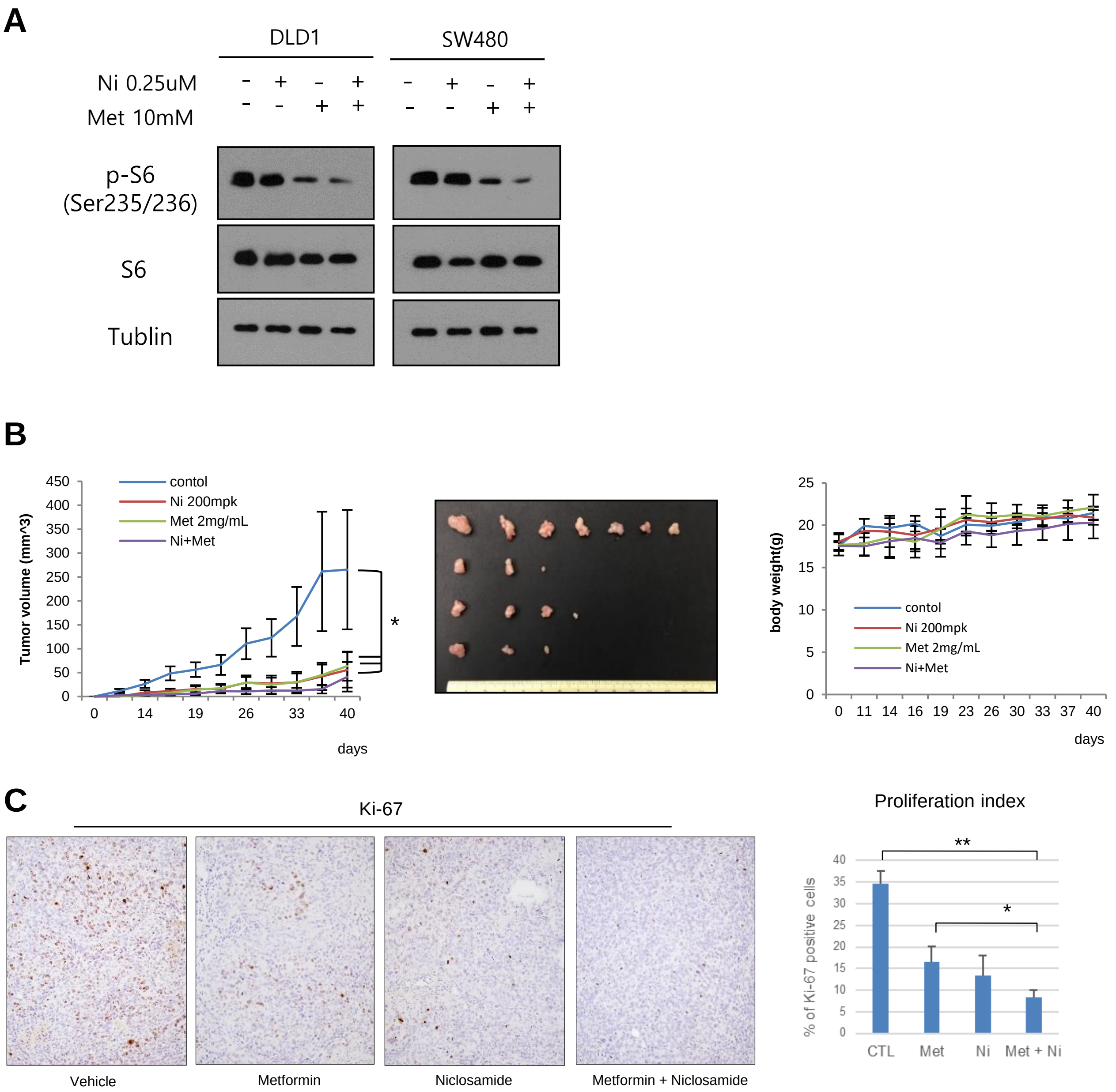

Figure S4. Metformin, in combination with niclosamide, suppresses mTOR activity and adenoma formation in APC-MIN mice.

A The CRC cells were treated with niclosamide (0.2 μM) or metformin (10 mM) or the combination, and mTOR activity was determined by pS6 and S6.

B SW480 CRC cells were inoculated into the flank of athymic nude mice prior to 24 h of treatment. Mice were treated daily (5 days a week) with vehicle or with niclosamide (200 mg/kg, P.O.) or with metformin (2 mg/mL, P.O.) or their combination. The tumor growth (left) and body weight (right) were monitored twice a week. Gross images of xenografted tumor mass for each group (middle).

C Xenografted tumor tissues of each group were formalin-fixed and paraffin-embedded sections were immunostained with Ki-67 (left panels). The images were taken from 10 randomly selected high power fields, and the proliferation index of Ki-67 positive cells were obtained using the ImageJ program (right).

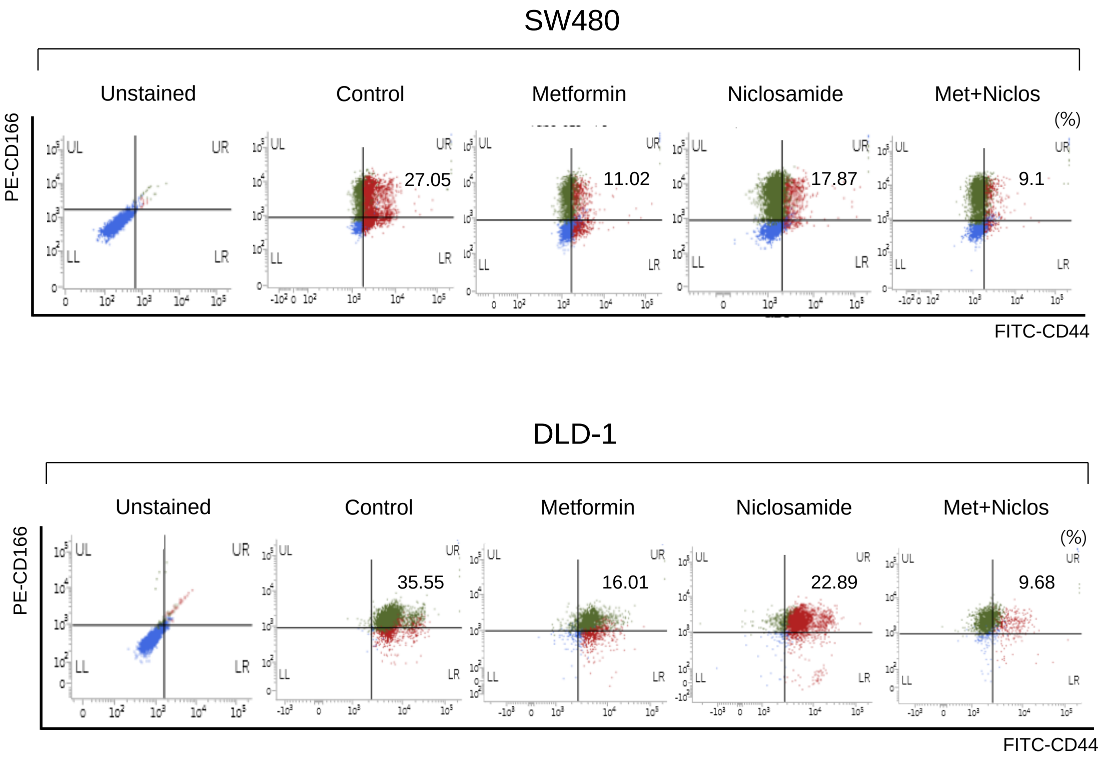

Figure S5. FACS Plot data

FACS with CD44 and CD166 markers. SW480 and DLD-1 cells were treated with metformin (10 mM), niclosamide (0.1 μM) and their combination.

Figure S6. Uncropped images of all the western blot data

Fig. 1B

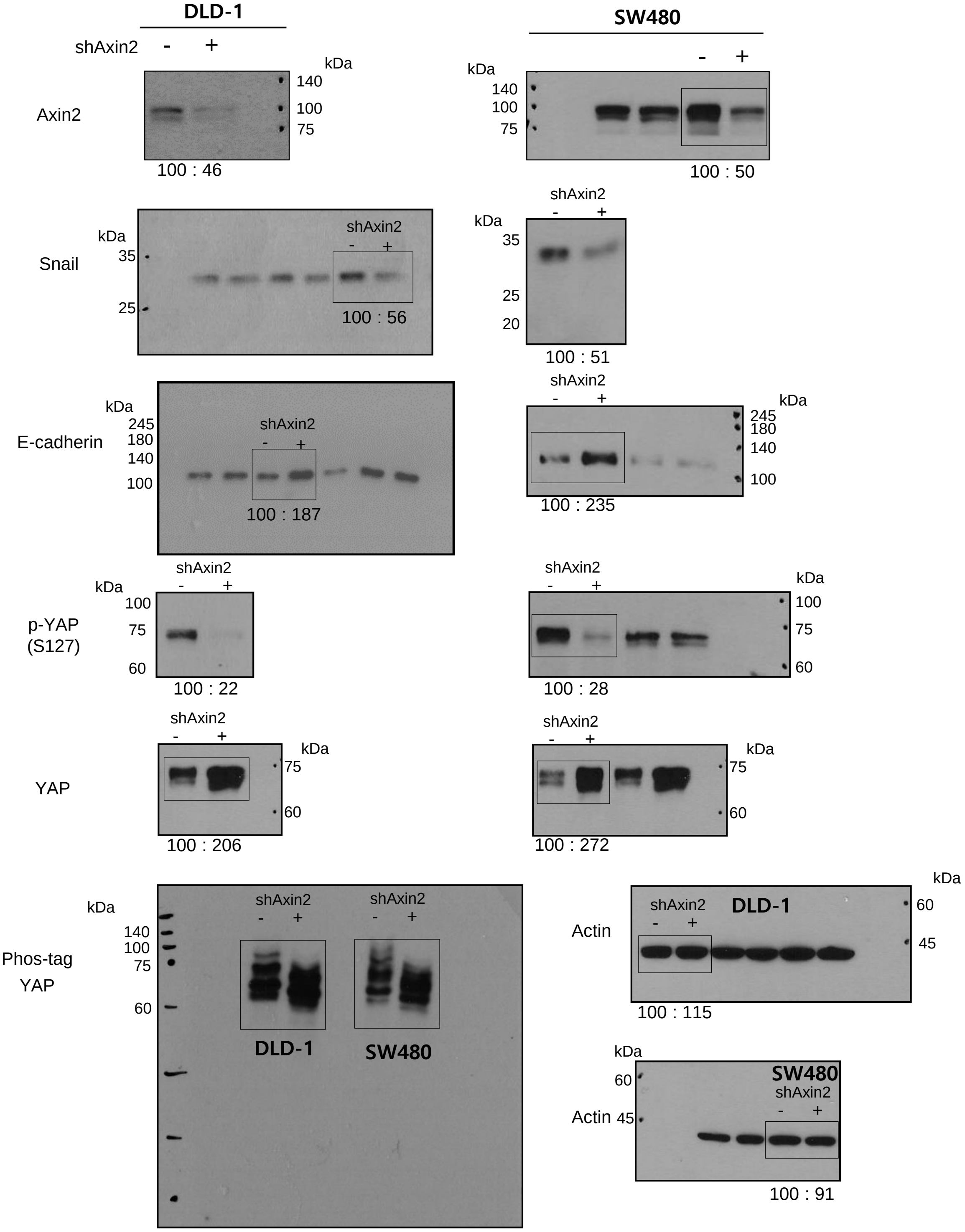

Fig. 1C

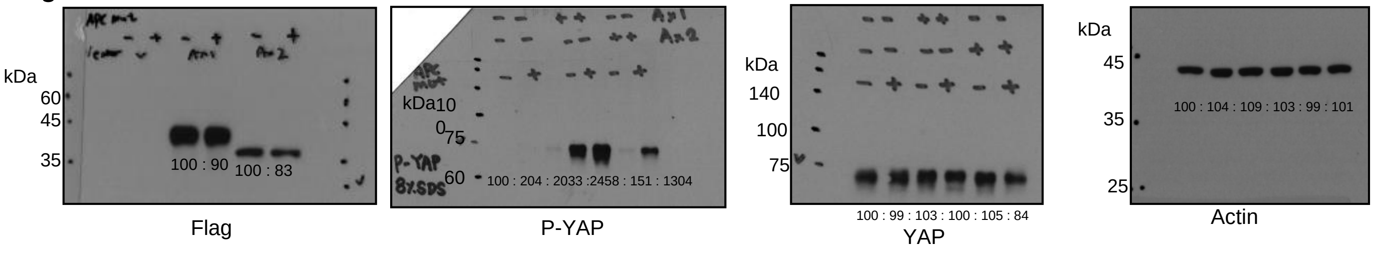

Figure S5. Continued

Fig. 2A

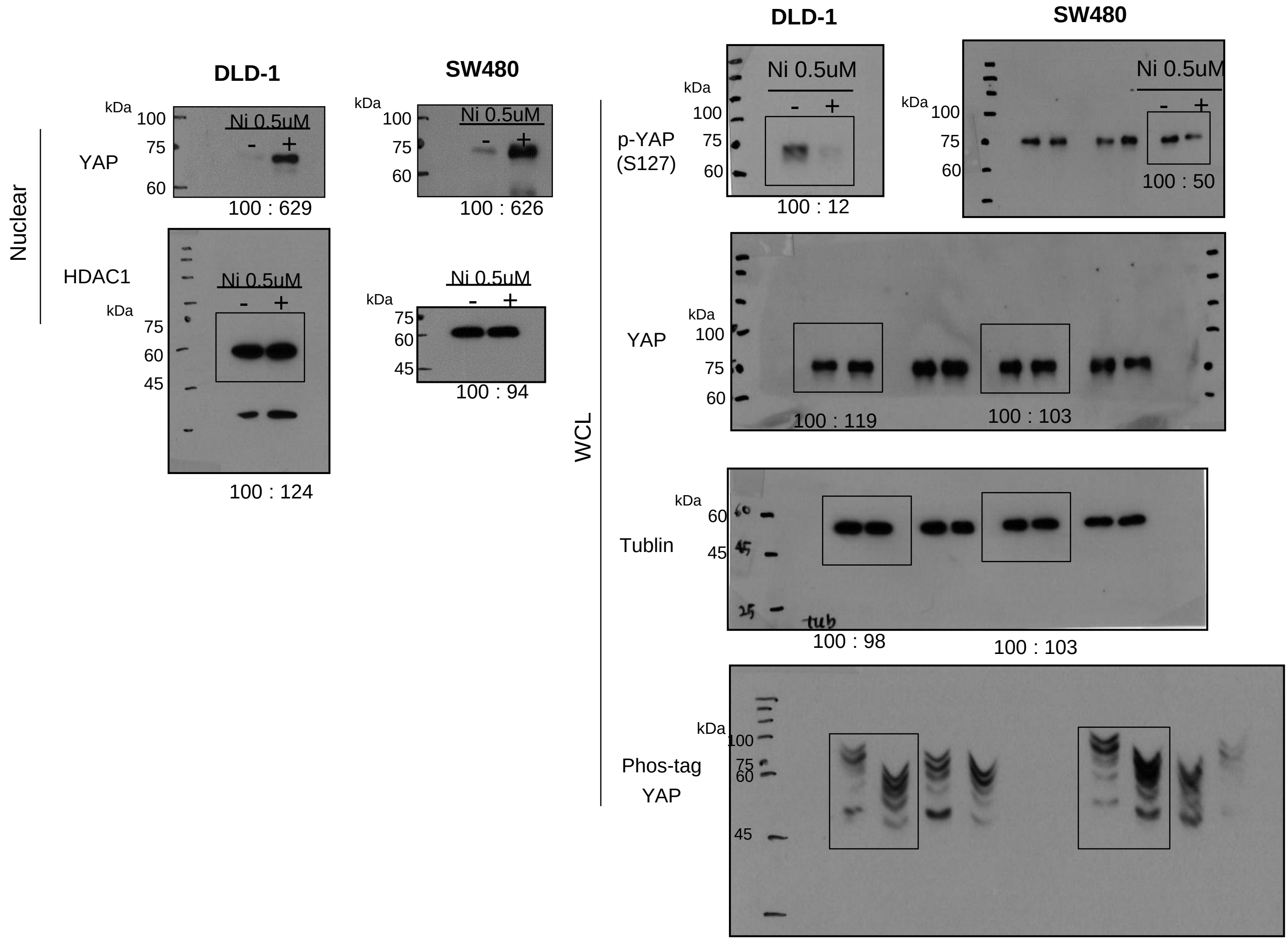

Fig. 2D

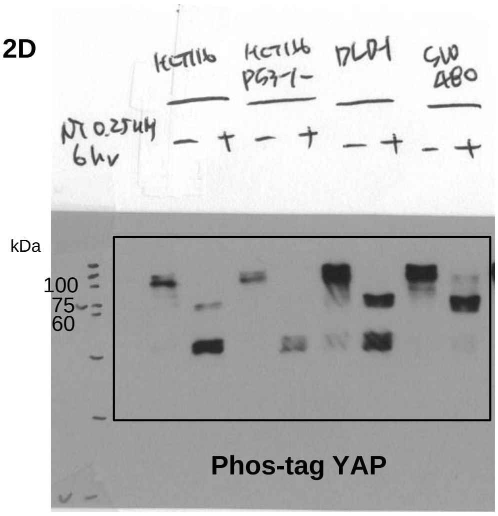

Figure S5. Continued

Fig. 3A

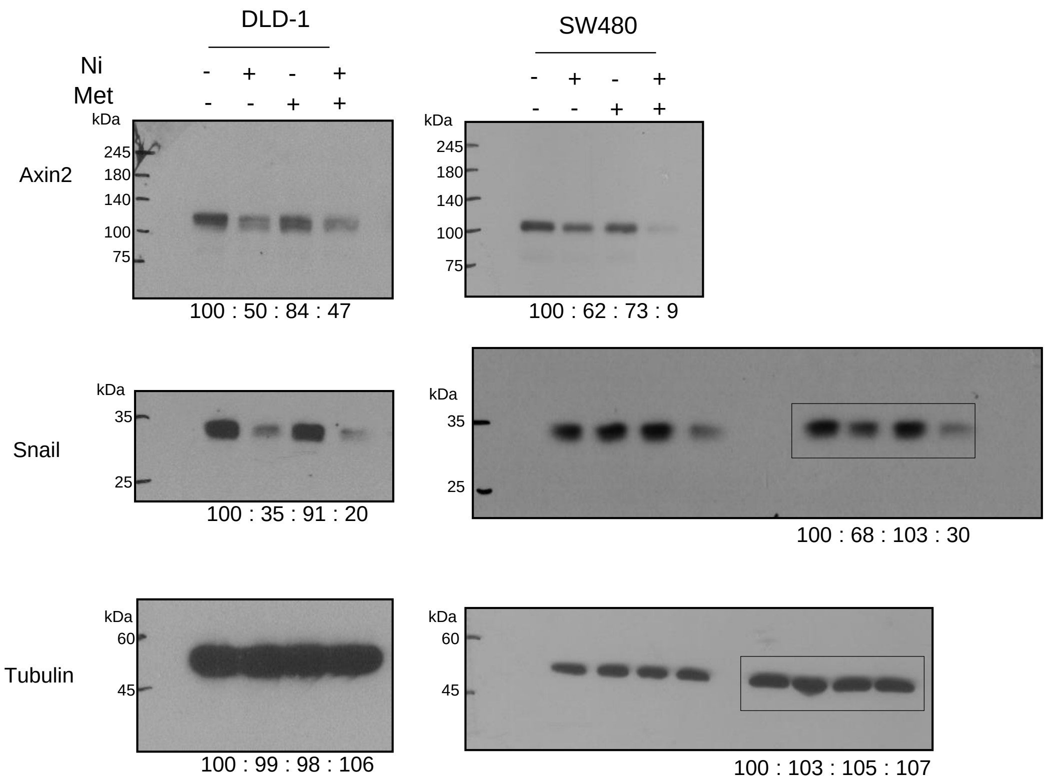

Fig. 3D

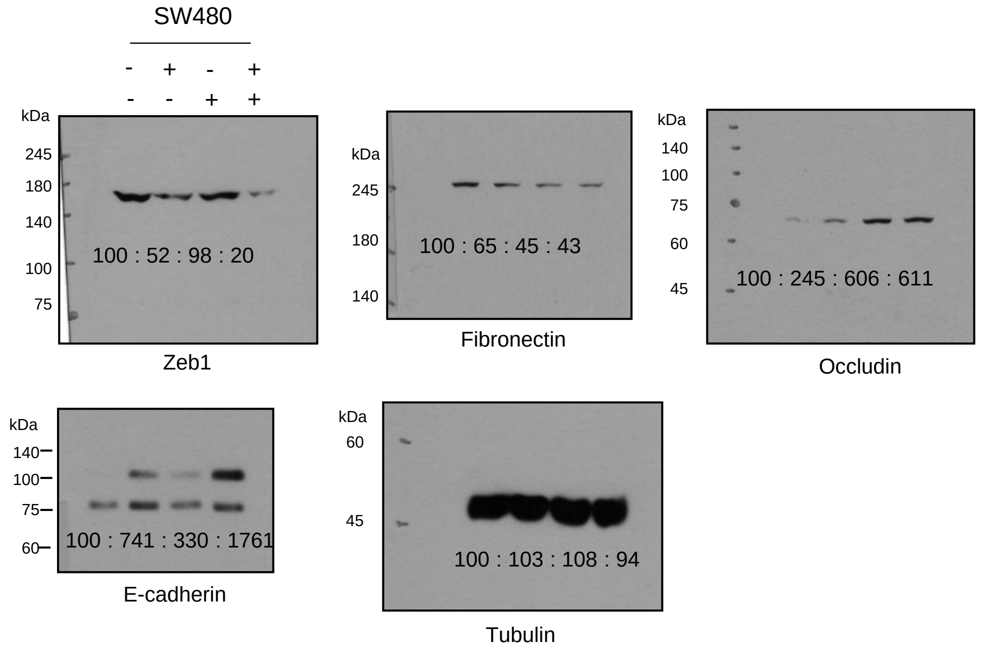

Figure S5. Continued

Fig. 4B

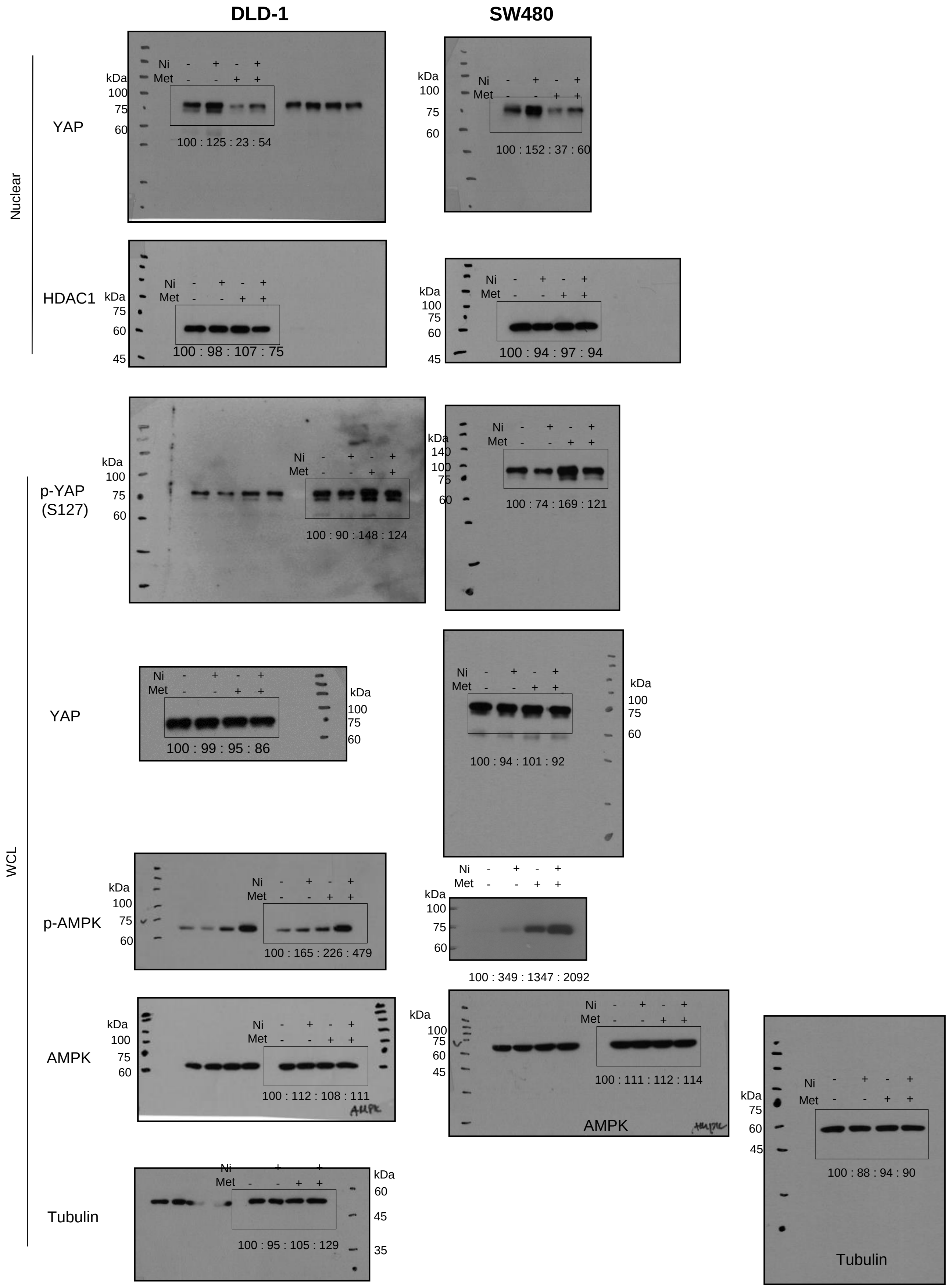

Figure S5. Continued

Fig. S1A

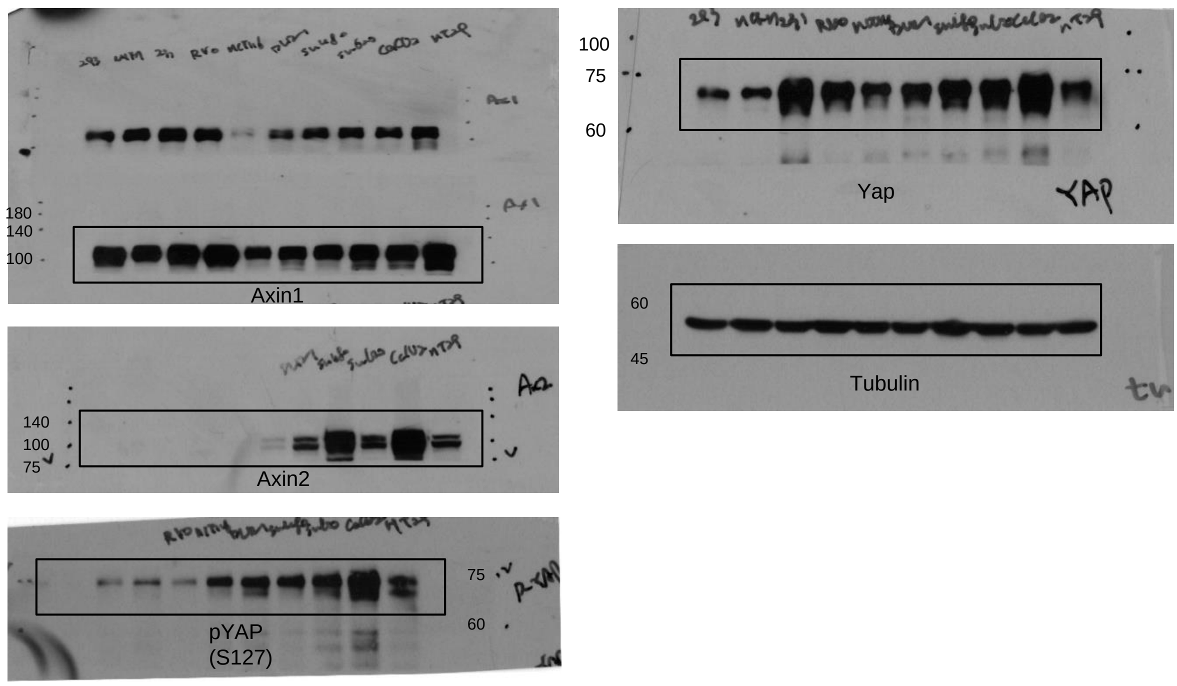

Fig.S2A

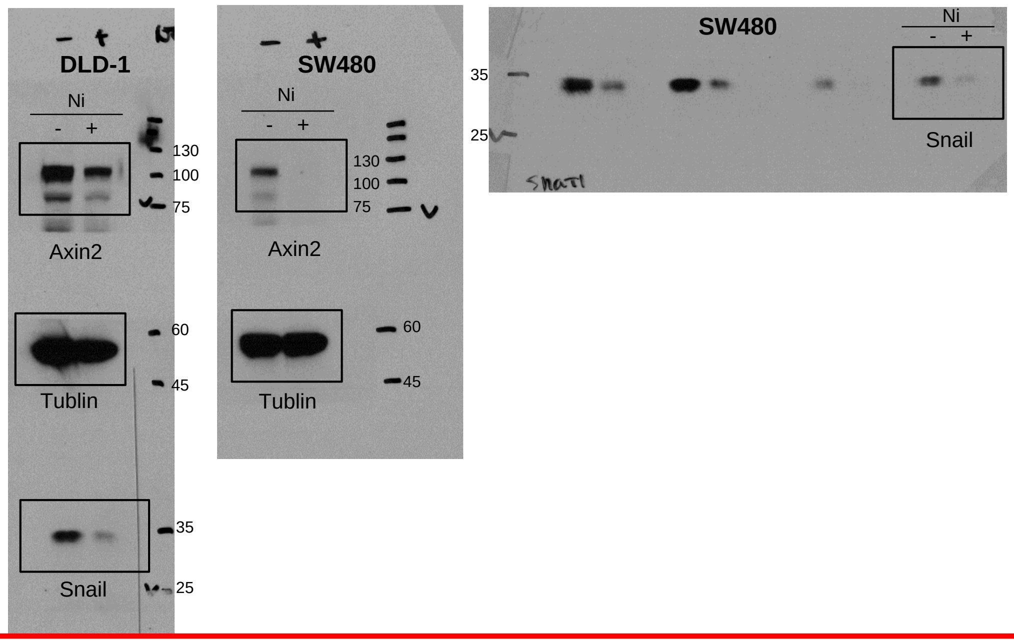

Fig.S2C

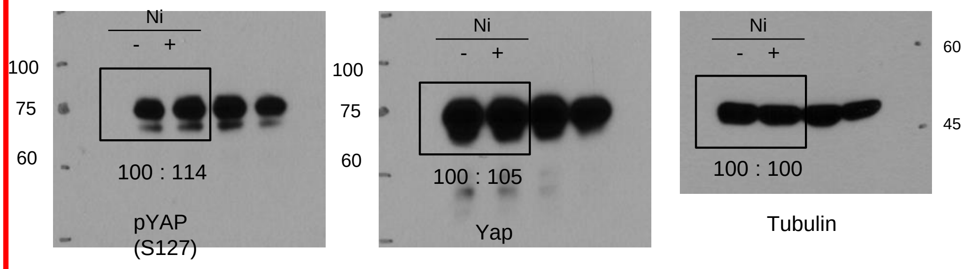

Figure S5. Continued

Fig.S3A

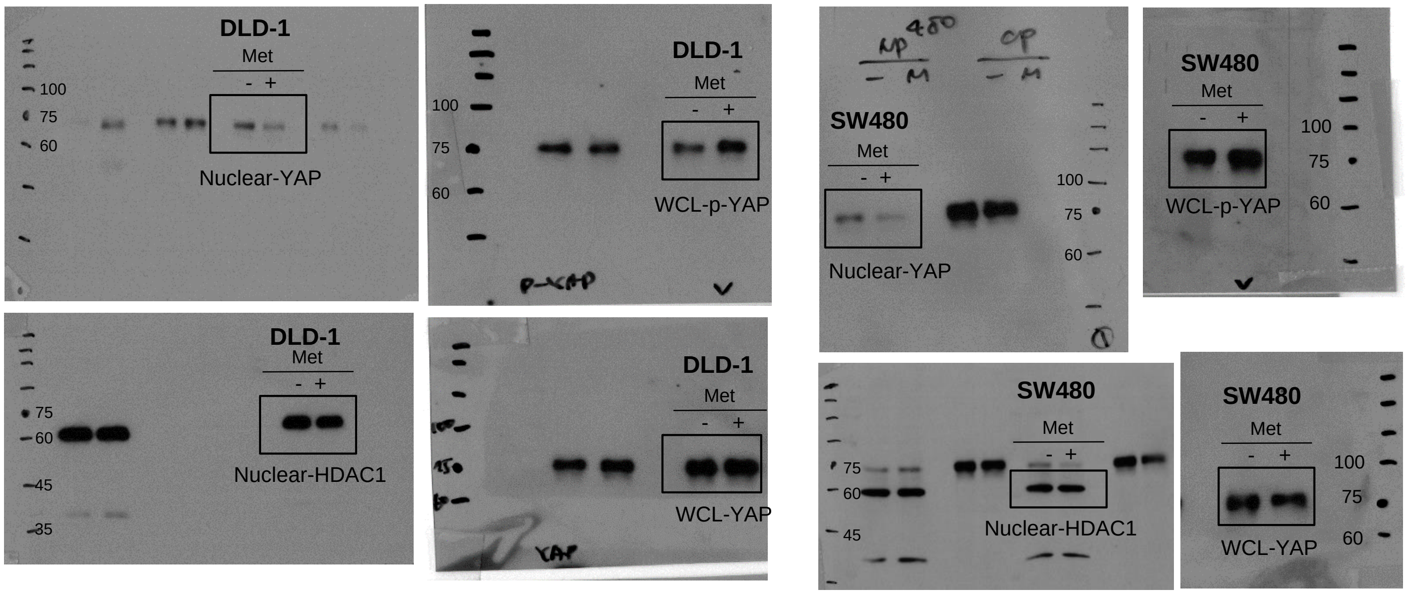

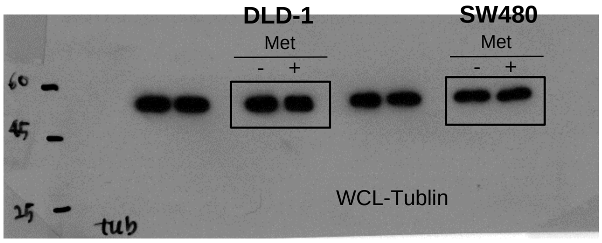

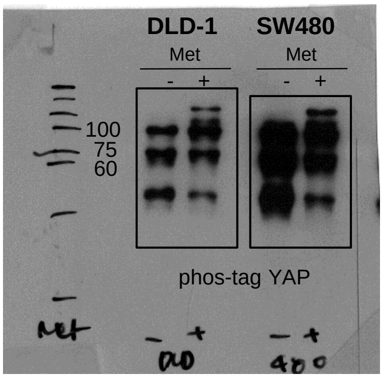

Fig. S3C

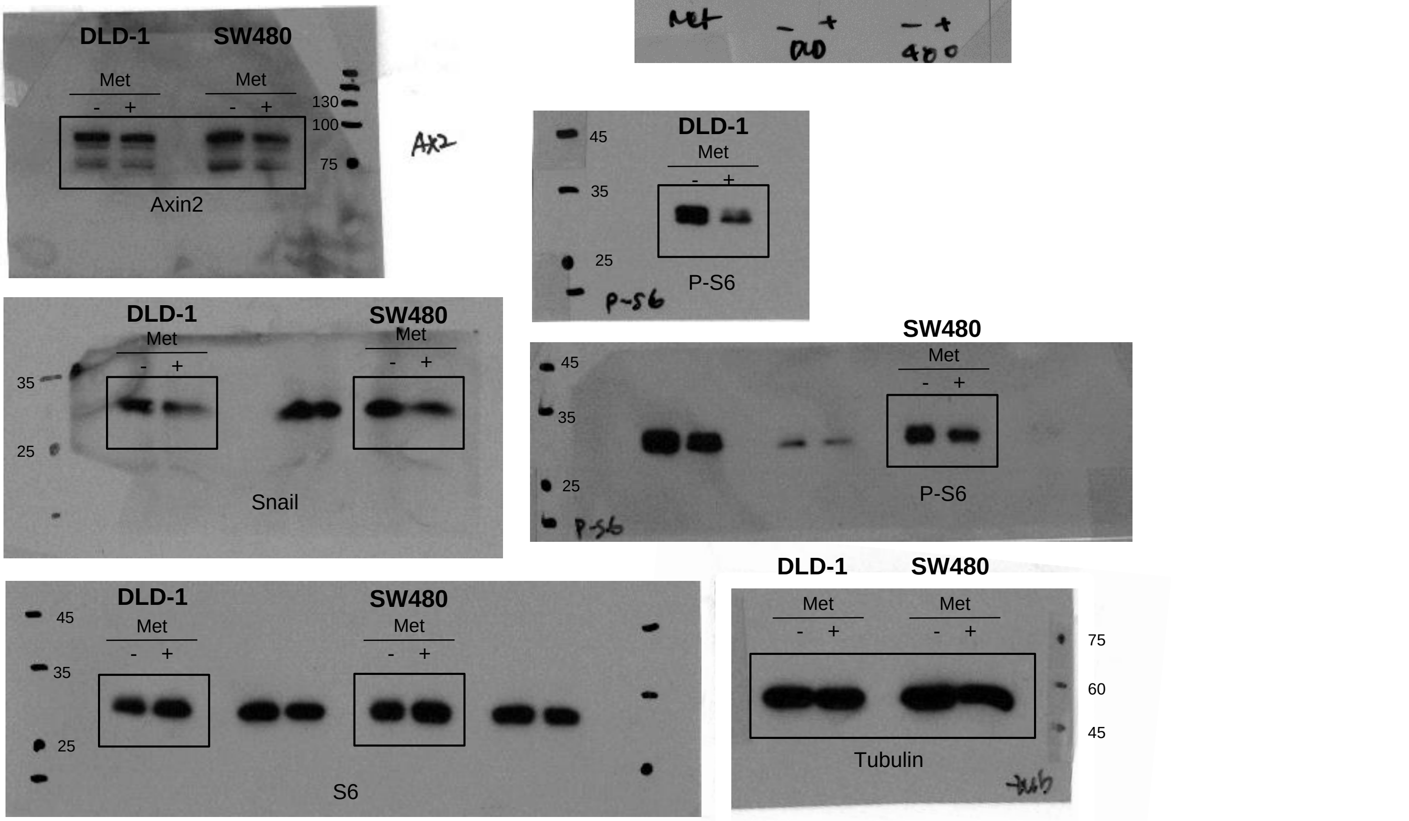

Figure S5. Continued

Fig. S4A

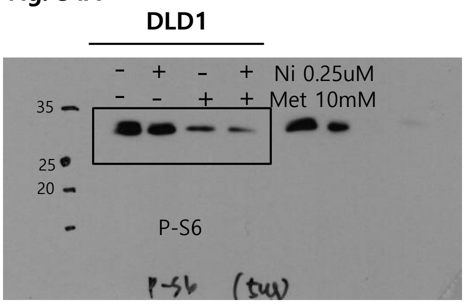

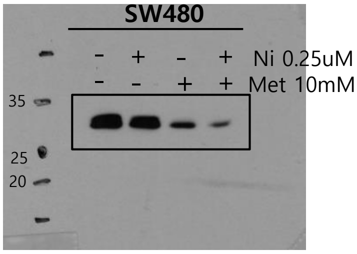

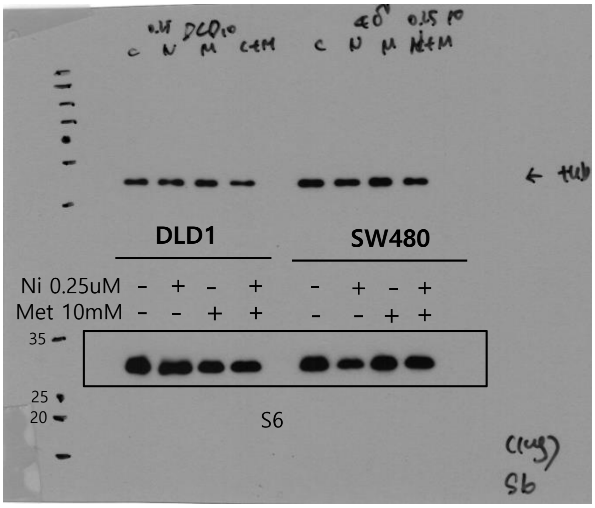

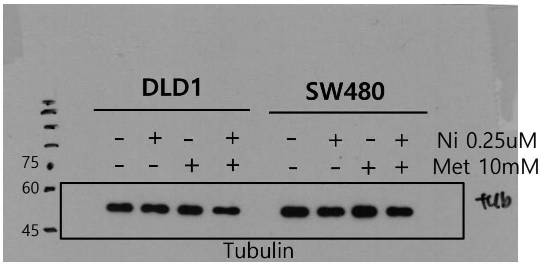

Table S1. Primer sequences used for Real-time quantitative PCR

Supplemental Table 1. Primer sequences used for Real-time quantitative PCR		
Genes	Foward	Reverse
E-cadherin	TGAGTGTCCCCCGGTATCCTC	CAGTATCAGCCGCTTTCAGATTTT
Claudin	GGCTGCTTTGCTGCAACTGTC	GAGCCGTGGCACCTTACACG
Occludin	CGGTCTAGGACGCAGCAGAT	AAGAGGCCTGGATGACATGG
Snail	TCTCTGAGGCCAAGGATCTC	CTTCGGATGTGCATCTTGAG
Zeb1	GCACCTGAAGAAGACCAGAG	TGCATCTGGTGTTCCATTTT
Fibronectin	CAGGATCACTTACGGAGAAACAG	GCCAGTGACAGCATACACAGTG
Axin2	AAGGGCCAGGTCACCAAAC	CCCCCAACCCATCTTCGT
CTGF	CAAAATCTCCAAGCCTATCAAGTT	CTCCACAGAATTTAGCTCGGTAT
GAPDH	TCCGCGGCTATATGAAAACAG	TCGTAGTGGGCTTGCTG AA
